# Enhancing Taxonomic Categorization of DNA Sequences with Deep Learning: A Multi-Label Approach

**DOI:** 10.3390/bioengineering10111293

**Published:** 2023-11-08

**Authors:** Prommy Sultana Hossain, Kyungsup Kim, Jia Uddin, Md Abdus Samad, Kwonhue Choi

**Affiliations:** 1 Computer Science, George Mason University, Fairfax, VA 22030, USA; 2 Department of Computer Engineering, Chungnam National University, Yuseong-gu, Daejeon 34134, Republic of Korea; 3 Artificial Intelligence and Big Data Department, Endicott College, Woosong University, Daejeon 34606, Republic of Korea; 4 Department of Information and Communication Engineering, Yeungnam University, Gyeongsan-si 38541, Gyeongsangbuk-do, Republic of Korea

**Keywords:** convolutional autoencoder, variational autoencoder, extreme learning machine, DNA sequencing

## Abstract

The application of deep learning for taxonomic categorization of DNA sequences is investigated in this study. Two deep learning architectures, namely the Stacked Convolutional Autoencoder (SCAE) with Multilabel Extreme Learning Machine (MLELM) and the Variational Convolutional Autoencoder (VCAE) with MLELM, have been proposed. These designs provide precise feature maps for individual and inter-label interactions within DNA sequences, capturing their spatial and temporal properties. The collected features are subsequently fed into MLELM networks, which yield soft classification scores and hard labels. The proposed algorithms underwent thorough training and testing on unsupervised data, whereby one or more labels were concurrently taken into account. The introduction of the clade label resulted in improved accuracy for both models compared to the class or genus labels, probably owing to the occurrence of large clusters of similar nucleotides inside a DNA strand. In all circumstances, the VCAE-MLELM model consistently outperformed the SCAE-MLELM model. The best accuracy attained by the VCAE-MLELM model when the clade and family labels were combined was 94%. However, accuracy ratings for single-label categorization using either approach were less than 65%. The approach’s effectiveness is based on MLELM networks, which record connected patterns across classes for accurate label categorization. This study advances deep learning in biological taxonomy by emphasizing the significance of combining numerous labels for increased classification accuracy.

## 1. Introduction

The high mobility of the global population and the effects of globalization have led to the emergence and rapid spread of new viruses and bacteria, similar to the COVID-19 pandemic. It is crucial to detect and identify infections early to prevent outbreaks and facilitate therapeutic development [1]. The categorization of Deoxyribose Nucleic Acid (DNA) sequences holds significant importance within the field of computational biology. In the event of a patient contracting a viral infection, it becomes necessary to conduct sequencing of samples and genomes to get insights into the virus’s source and facilitate the development of vaccinations. Quickly and accurately identifying the pathogen’s phylogenetic tree is essential.

To classify DNA sequences according to their taxonomy, researchers in this paper have developed two types of deep learning (DL) algorithms. Sequences and domain labels used in this research were obtained from the National Center for Biotechnology Information (NCBI) database, which houses a large amount of genomic sequence data. Machine learning algorithms are utilized to address the exponential growth in the number of DNA sequences.

DNA functions as the fundamental genetic material for all living organisms and is made up of a set of four nucleotides, namely adenine (A), cytosine (C), guanine (G), and thymine (T). Every virus possesses its own distinct DNA, and the specific arrangement of nucleotides within the DNA molecule is responsible for conferring its unique properties [1]. DNA has the ability to exist in two distinct forms: single-stranded and double-stranded. In the double-stranded form, nucleotides on one strand are able to form pairs with their complementary counterparts on the opposing strand. RNA is a molecule that demonstrates close connection and has the ability to exist in either a one-stranded or two-stranded conformation. In RNA viruses, uracil (U) is present instead of thymine (T). The genome consists of a specific arrangement of nucleotides, namely adenine (A), cytosine (C), guanine (G), and thymine (T) for DNA viruses. For bacteria, adenine (A), cytosine (C), uracil (U), and guanine (G) exist.

The process of categorizing domain labels in the analysis of DNA sequences entails the application of the Word2Vec algorithm to extract relevant features from the unprocessed DNA sequence. The aforementioned characteristics are subsequently utilized as inputs for the Stacked Convolutional Autoencoder MLELM (SCAE-MLELM) and Variational Convolutional Autoencoder Multilabel Extreme Learning Machine (VCAE-MLELM) models. These models are then compared to other deep learning (DL) models, including CNN-Bidirectional LSTM [1] and DeepMicrobe [2], with respect to accuracy and various other metrics. The aim of this study is to identify the most effective encoding and architecture methods for extracting features from DNA sequences and achieving an accurate classification of taxonomy labels. The classification of domain labels in DNA sequence analysis involves utilizing characteristics extracted from the raw DNA sequence using the Word2Vec algorithm. These features are then inputted into the Stacked Convolutional Autoencoder-Multilabel Extreme Learning Machine (SCAE-MLELM) and Variational Convolutional Autoencoder—Multilabel Extreme Learning Machine (VCAE-MLELM) models, which are compared with other DL models such as CNN-Bidirectional LSTM [1], and DeepMicrobe [2] in terms of accuracy and other metrics. The objective is to determine the optimal encoding and architecture for extracting features from DNA sequences and accurately classifying taxonomy labels.

The sequence classification challenge involves classifying the taxonomy and establishing phylogenetic groups for a set of genomics sequences, such as DNA or RNA sequences. This problem is important in bioinformatics as it relates to specific tasks like virus sub-typing, haplogroup identification, or predicting the group to which a new sequence belongs.

Various approaches have been used in this field, including alignment-based and alignment-free methods. The objective of this study is to introduce a methodology that outperforms current contemporary techniques in terms of both effectiveness and accuracy. The key contributions of this study are as follows.

- The proposed strategy addresses the issue of uneven sequence lengths in DNA or RNA strands by dividing them into equal lengths of 3000 nucleotides per sequence. Additional nucleotides are added to strands that cannot reach the desired length to ensure uniformity.
- The sequences are then encoded using one-hot encoding and transformed into 4-mer color platelet images, where each unique 4-mer sequence is assigned a distinct color. The split-sequence data overlap by 50% to create the 4-mer relationship matrix, which captures the position and relationship between 4-mers.
- This data is fed into a neural network for taxonomy classification. SCAE and VCAE structures extract detailed feature vectors, and a series of Multilabel Extreme Learning Machines generate soft classification scores and hard labels for unsupervised DNA sequences.

## 2. Literature Review

Scientists used to categorize organisms according to their form and structural characteristics using morphological techniques. However, modern taxonomy relies on DNA data and several methods, including as sequence alignment and DNA barcoding, to identify and classify species [3]. Organisms are classified into kingdoms, phyla, classes, orders, families, genera, and species in addition to these fundamental divisions. Humans, for instance, are classified into haplogroups based on shared ancestry. Proteins are encoded by DNA or RNA sequences, which are known as genomics. DNA contains the instructions needed to make proteins. Reading or sequencing DNA is the process of determining the nucleotide order on a DNA strand. It is often achieved by breaking the DNA strand into smaller fragments and stitching them together. Next-generation sequencing methods, which reduce sequencing costs and boost speed but require more processing power, are used to read shorter fragments. the sequence assembly effort. Alignment-free sequence classification techniques have therefore gained popularity.

A multitude of alignment-free sequence comparison algorithms have been developed in recent years, such as conditional Lempel-Ziv [4] and Kolmogorov complexity [5], measure representation [6], Markov model comparisons and frequent substring lengths [7,8], which divides the genome into regions that represent a system that is evolving over time with hidden states. Base-base correlation [9], spectral distortion [10], primitive discrimination substrings [11], Burrows-Wheeler similarity [12], normalized central moments, nearest-neighbor interactions [13], subword composition [14], prefix codes [15], information correlation [16], the context-object model [17], and spaced word frequencies [18].

These algorithms are used in sequence classification (e.g., COMET [19]), sequence composition (e.g., COMET [19]), restriction enzyme site distributions (the ‘natural vector’ for viral genome and proteome classification [20]), and neural networks that use genomic cepstral coefficient characteristics to distinguish deadly viruses [21]. Alignment-free methods provide an option for comparing sequences, making it possible to analyze and classify a variety of genomic data efficiently.Methods utilizing k-mer frequencies, which examine the patterns of occurrence for substrings with a length of k, are commonly employed for sequence comparison without alignment. These methods were invented by Blaisdell, who created phylogenetic trees for other DNA sequences [22] and alpha- and beta-globin genes [23].

Further investigation into k-mer bias patterns has revealed connections between these patterns and many DNA sequence characteristics, including repair mechanisms, mutations, and DNA/RNA structure. It has also been shown that k-mer proportions can function as genomic signatures, allowing sequences from the same organism to be distinguished from one another.

In supervised classification, k-mer frequency vectors have been used. Nonetheless, it is more frequently seen in small datasets. The field of influenza subtyping [24], viral fragment classification, HPV genotype prediction [25], taxonomic grouping of bacterial and eukaryotic genomes [17], identification of microbial DNA sequences [26], differentiation between the genomes of *E. coli* and yeast [27], classification of bacterial genome fragments [25], classification of splicing-related sequence snippets [28], and classification of archaeal and bacterial groups [29] have all been observed applications of these techniques.

These k-mer-based approaches offer an effective means for sequence comparison and classification, leveraging the abundance and distribution patterns of k-mers to gain insight into genomics relationships.

Researchers have employed various combinations of deep learning techniques to extract high-level features from sequences that represent DNA or RNA. For example, Ref. [30] employed Long Short-Term Memory (LSTM) to process these features from binary-encoded DNA sequences, demonstrating that this approach outperforms current state-of-the-art methods. Abbas et al. [31] employed five distinct feature encoding techniques to extract relevant features from RNA sequences. They used SHapley Additive exPlanations to select the best features among the extracted ones then XGBoost, a machine learning classifier, was employed in the model. Additionally, an optimization method called “Optuna” was utilized to efficiently determine the best hyperparameters for the model. Rehman et al. [32] represents DNA sequences numerically using a single encoding technique. After that, a series of convolution layers process the numerical data in order to extract low-level features. The capsule network uses these properties in addition to extracting intermediate and high-level features that are used to categorize 6 mA sites. For shallow feature extraction, each of the contextual feature vectors derived from the encoding methods is independently processed via a number of neural network layers [33].

In this study, we conducted a comparative analysis between CNN-Bidirectional LSTM [1] and DeepMicrobe [2], to categorize DNA sequences based on 4 species taxonomic rank. While prior methods required a carefully curated taxonomic tree, DeepMicrobe addresses the challenge of integrating genomic assemblies of uncultivated species into a taxonomic reference database. DeepMicrobe has created a deep learning-based computer architecture that goes beyond the constraints of conventional techniques. It has been demonstrated to be more accurate in estimating abundance while being more successful in identifying species and genera. It was trained on genomes of the gut microbiome, which simplified the study of uncharacterized metagenomic species and may have yielded new signatures in inflammatory bowel diseases. Conversely, CNN-Bidirectional LSTM, which has an architecture modeled after AlexNet, performs transfer learning and makes use of Artificial Neural Networks (ANN). It also includes a bidirectional long-short-term memory (BiLSTM) layer, which takes temporal characteristics into account during processing.

## 3. Dataset Collection

The entire DNA/Genomics sequence of viruses such as cherry virus, cereal yellow dwarf, human fecal virus, and others was acquired from “the National Center for Biotechnology Information (NCBI)”, a public nucleotide sequence database. DNA sequence data are in FASTA format. The sequence varies in length from 8 to 76,000 nucleotides. The distribution of hosts in the class, together with the corresponding sample sizes, is as follows: 13.5% for plants, 20.6% for fungi, 29.7% for humans, and 36.3% for bacteria.

It is evident from the dataset that there is a problem with the dataset being skewed. To solve this problem, SMOTE (Synthetic Minority Oversampling Technique) [34] might be used. The host and fungus DNA sequences of plants are limited in our database. The SMOTE method might be used to create synthetic samples for minority classes, such as fungi and plants, that closely matched the majority class. The SMOTE algorithm employs a random selection process to choose an instance from the minority class. Subsequently, it identifies the k nearest neighbors belonging to the same minority class. Synthetic examples are created by combining two selected instances using a convex combination. This methodology can generate a fabricated scenario for the underrepresented categories.

### 3.1. Preprocessing Dataset

**Figure 1:**
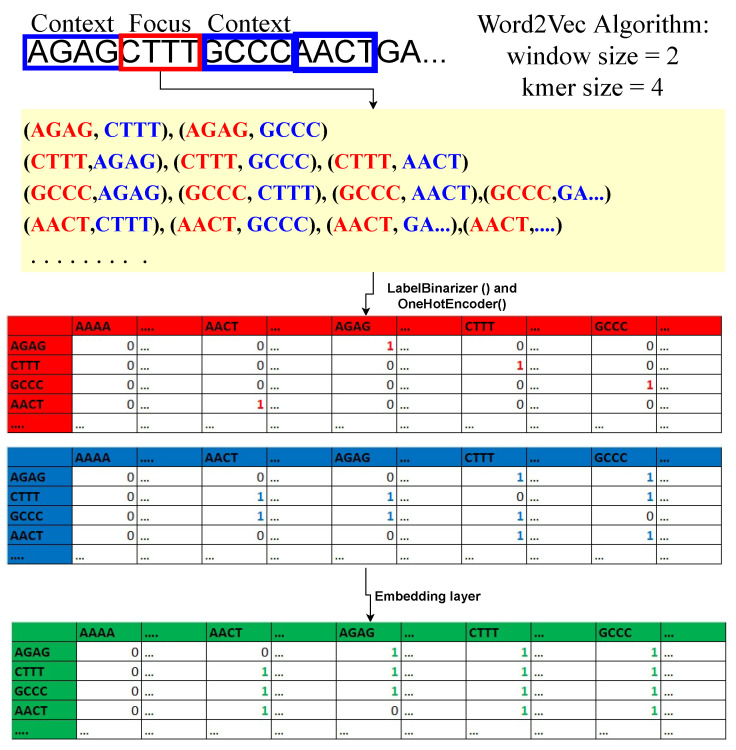
Formation of dense relationship feature vector matrix from the 4-mer sequence with sliding window = 1.

Regarding machine learning and deep learning, pre-processing is the most crucial step whenever numerical input is used instead of categorical. The genomics sequence in the chromosome data is categorized. There are several strategies for converting category data to numerical data. The process of transforming nucleotide categorical data into a numerical form is known as encoding. Label encoding, one-hot encoding, and K-mer encoding are utilized to encode the DNA sequence in this study. As shown in Figure 1, the DNA sequence gives an index value (AAAA-1, AAAC-2, AAAG-3, and AAAT-4) to each 4-kmer sequence nucleotide using label encoding and then transforms it to one-hot encoding.Using the LabelBinarizer() and OneHotEncoder() functions in Python, the complete DNA sequence is transformed into an array of integers.

By producing 4-mers for the raw DNA sequence, the k-mer encoding approach converts it into an English-like statement. Each DNA sequence is turned into a K-mer of size 4, and all of the K-mers produced from the sliding window set to 1 are concatenated to make a sentence of 3000 in length. These DNA sequences were classified using a natural language processing (NLP) approach. During this investigation, the sequence embedding layer was operationalized to effect a transformation of the K-mer phrase into a matrix composed of dense feature vectors.

In the embedding layer of the sequence, word2Vec algorithm is used. Commonly used in NLP [35] to learn features from a set of k-mer sequences. Here k-mer sequences are mapped to a vector of real numbers. For example; “AAAA” = [0.148,0.4756,…,1.248]. The important feature in k-mer sequence embedding is that similar k-mers in the semantics sense (the digital information of the linear genetic code) have smaller Euclidean cosine distances between them compared to k-mer sequences that have no semantic relationship. The model under consideration comprises a sequence embedding layer, which is a neural network consisting of an input layer, a hidden layer, and an output layer. The determination of the training matrices for sequence embeddings involves the selection of a hyperparameter, namely the window size (w) of the context k-mer. The minimum size of w must be 1, as the algorithm cannot function without context k-mer. For example, if w=1 and k−mer=3 for a sample DNA sequence “**AAT**CGCTTTAGCTA…”. The bold 3-mer is the focused sequence, and one 3-mer sequence to the left and right of the focus sequence is the context sequence. Therefore, with this logic, we can build data points and a dense relationship feature vector matrix for a DNA sequence, as shown in Figure 1. The focal point of the analysis is the 4-mer sequence matrix, with an emphasis on the red table. The purpose of this structure is to maintain the location of the focal sequence within the strand. Second, the blue table is the context 4-mer sequences matrix. This preserves the relationship between the focus 4-mer sequence and context 4-mer sequences. Lastly, the concatenation of the focus and context matrices is done to form the relationship-dense feature vector matrix. The embedding dimension (hidden layer) is set to 2 to later plot the 4-mer sequences in the data space and see whether similar 4-mer sequences cluster. Softmax activation, a linear function, is used in the output layer of the neural network. The input dimension is equal to the total number of unique 4-mer sequences. Keras and tensorflow are used to train the network.

### 3.2. Multilabel Data Representation

Each data instance for a given number of classes, denoted by *C*, is a subset $F_{i}=f_{i1},f_{i2},f_{i3},\ldots ,f_{in}\left(i=1,\ldots ,N\right)$ related to a vector $O_{i}=o_{i1},o_{i2},\ldots ,o_{iC}$ [36]. It is possible for an instance to belong to more than one class simultaneously. The values in the output vector are represented in binary format, with 1 indicating that the sample belongs to a particular class category and 0 indicating that it is not similar to that class category. The utilization of several class labels on a single instance is feasible in this scenario, unlike with signal-label data, where it is impossible. A label set refers to every possible combination of class labels. More details regarding the representation of multiple labels will be discussed in subsequent sections.

## 4. Proposed Models

**Figure 2:**
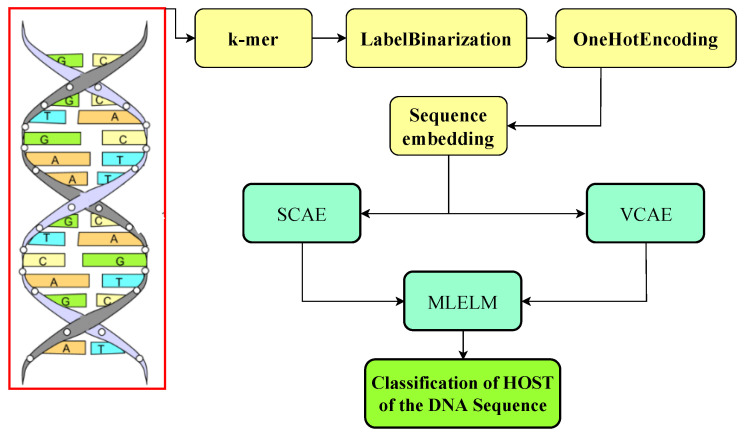
Overview of the proposed system.

The researchers in this paper introduced two deep-learning models to address the challenge of identifying the host from which a DNA sequence originates. The first model combines a stacked convolutional autoencoder (SCAE) with MLELM. The second model utilizes a variational convolutional auto-encoder (VCAE) within the MLELM framework for classification based on one-hot encoding input data. The researchers conducted a series of experiments using several deep learning models. The study’s findings indicated that the optimal model for SCAE-MLELM was a completely interconnected SCAE combined with MLELM. This model demonstrated superior soft categorization and score estimation performance across various classes. The utilization of a convolutional autoencoder facilitates the extraction of the spatial organization inherent in DNA sequences, hence enhancing computational efficacy and performance through the identification and representation of latent associations among data attributes. The extracted features are subsequently inputted into two MLELMs, with the initial model generating probabilistic labels and the subsequent model establishing a relationship between deterministic labels and probabilistic labels. Unseen data is assigned hard labels based on the projected ratings.The VCAE-MLELM architecture is kept simple, with fully connected convolutional and deconvolutional layers, and mean and variance nodes in the autoencoder bottleneck. A comprehensive overview of the proposed system is provided in the following sections, and the research design is illustrated in Figure 2.

### 4.1. Stacked Convolutional Autoencoder (SCAE)

Autoencoders are neural networks that compress input data into a lower-dimensional representation and then reconstruct it. Convolutional Neural Networks (CNNs) have convolutional, pooling, and fully connected layers. Autoencoders operate in an unsupervised manner, whereas CNNs are feedforward networks.

The encoder in an autoencoder maps the input to hidden nodes using a transfer function. The decoder reconstructs the original input from the hidden representation. The weights are updated through backpropagation. The proposed model includes convolutional layers and ReLU activation functions, forming a Stacked Convolutional Autoencoder (SCAE) to capture hierarchical features.

The convolutional layer in the bottleneck position serves to flatten the data, which are then sent into a fully connected layer. The feature vector is used for the purposes of analysis and prediction by multilabel extreme learning machines. The encoder and decoder are configured to reduce the amount of error when reconstructing the input data.

**Figure 3:**
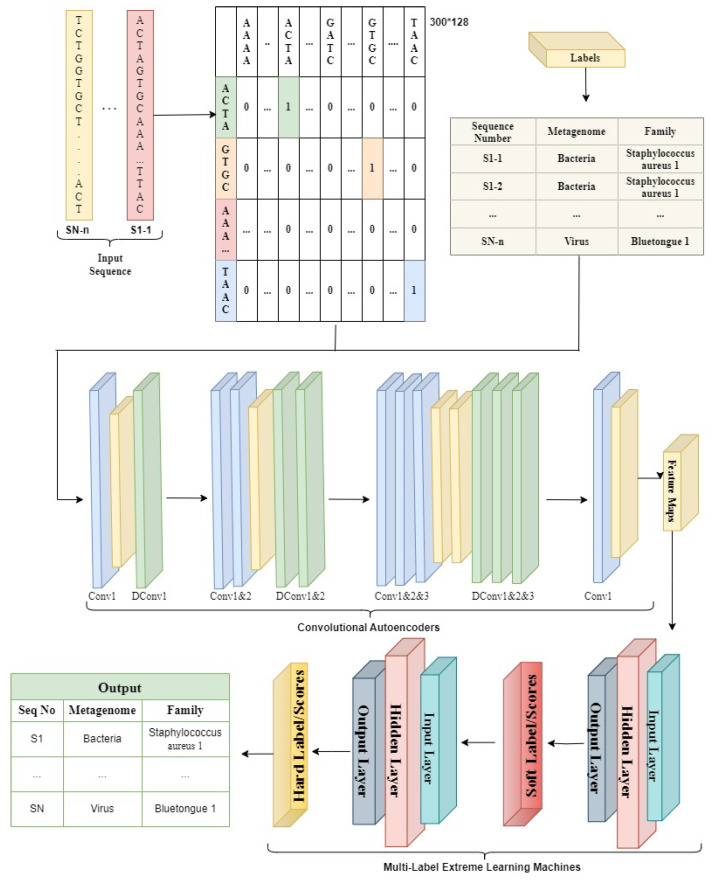
Proposed system workflow.

Figure 3 illustrates the structure of an auto-encoder, while CNNs have a different architecture comprising convolutional, pooling, and fully connected layers. The purpose of autoencoders is to learn a compressed representation of input data, whereas CNNs are primarily used for tasks such as image recognition and classification.

The proposed model employs convolutional layers with rectified linear unit (ReLU) activation functions, in addition to incorporating max-pooling layers inside the encoder portion. This choice of a convolutional autoencoder is made to improve computational complexity and overall performance [37]. The encoder component of the model employs a non-linear transformation to convert the input vector into a lower-dimensional hidden representation. Subsequently, the decoded part reconstructs the original input from this hidden representation. This process is repeated, forming a Stacked Convolutional Autoencoder (SCAE) that effectively captures hierarchical features in the data. The encoder and decoder structures are symmetric, allowing for the extraction of low-dimensional hierarchical features [38].

### 4.2. Variational Convolutional Autoencoder (VCAE)

A variational autoencoder (VAE) models observations in latent space using probability distributions instead of single values. By representing each latent property as a distribution, we can capture multiple alternative values for each characteristic. This approach allows for a more flexible and descriptive encoding of input data. The encoder model in VAE generates probability distributions for each latent property, while the decoder model reconstructs the input based on random samples from these distributions. This probabilistic representation ensures a smooth and continuous latent space, enabling similar values to produce similar reconstructions.

In a VAE encoder model, instead of directly producing values for the latent state, it generates parameters representing distributions for each dimension in the latent space. The mean and variance vectors are outputted to characterize these distributions based on the assumption of a normal prior. To simplify the model, we assume a diagonal covariance matrix with non-zero values only on the diagonal, representing the association between dimensions. The decoder model then reconstructs the input by sampling from these distributions. To enable backpropagation during training, a reparameterization technique is used, involving sampling from a unit Gaussian and adjusting the samples based on the mean and variance of the latent distribution. Additionally, the logarithmic transformation followed by an exponential is often used to handle negative values in the learned variance.

### 4.3. Extreme Learning Machine (ELM)

MLELM is a three-layer architecture consisting of input, hidden, and output layers. The weights linked to the input layer and biases are initially assigned random values and stay constant during the learning process, with the learning occurring only inside the hidden layer.The activation function ϑ is applied to calculate the output of each hidden node. The model weight matrix ϖ is obtained iteratively during the training phase, allowing class prediction in the testing phase. The proposed system combines the ELM network’s topology with encoded features from SCAE for Multilabel classification. A threshold determines the hard labels based on the score achieved, with the drawback of potential misclassification. The reduced number of input nodes in MLELM due to SCAE results in a compact weight matrix and a smaller hidden layer. The generation of soft classification label scores is followed by their utilization as input for the second MLELM model to make predictions for target labels without needing a predetermined threshold. The discrete class labels are obtained by converting the final score using a calibrated threshold.

## 5. Experimental Setup

The study employed 117,405 full-length sequences that were sourced from the NCBI database as its dataset. The input data was split into training sets (70%), validation (10%), and testing (20%), with each sequence being broken into 3000 nt, 82,215 training sequences, 11,740 validation sequences, and 40,000 testing sequences were the end results of this.Throughout the training procedure, the difference between the desired label and the anticipated output was computed using the binary cross-entropy function. The loss function is used to modify the model’s weights.

To test several models, hyperparameters like filter type, size, number of layers, and embedding dimension were changed. The grid search cross-validation method was used to choose the ideal parameters. The ideal number of filters in each layer for each of the three models was discovered to be 128, 64, and 32. The experiment’s configuration included the following values: the filter size was set to 22, the training batch size was set to 100, the number of training epochs was set to 10, the embedding dimension was set to 32, and the K-mer size was set to four.

During the training phase, the input data is passed through the Stacked Convolutional Autoencoder (SCAE). The Adam optimizer is used with a learning rate set at 0.001 to train the encoder and decoder components within the SCAE. The primary objective of this training process is to minimize the Mean Squared Error between the input data and its reconstructed output. Training continues until a stopping criterion is met. In this case, training stops after 10 epochs if there is no improvement in the validation loss, which serves as a metric to prevent overfitting. The choice of this stopping criterion is influenced by factors such as dataset size, model complexity, available computational resources, and the model’s performance. The SCAE employs an iterative learning approach by using multiple layers and progressively reducing the number of features to improve its ability to recognize input data.

Next, in order to do soft-class prediction, the encoded attributes gathered from the SCAE are input into the Multilabel ELM network (MLELM). MLELM processes all input instances simultaneously while operating in batch mode, with the number of hidden layers determined by the input nodes. With MLELM, weights learned from the hidden layer via the variable ϖ are used to compute class scores for encoded training data. The weighted outputs of the hidden nodes are added to produce these scores. The second MLELM network receives the expected scores after that, enhancing the predictions by comparing the class scores to the actual class labels. To extract the weights for the hidden layer in the second MLELM network, just one operation is needed. The test data is then autonomously put into the SCAE network following the training of the MLELM and SCAE networks. The procedure entails the creation of encoded attributes, which are then sent across MLELM networks.The hard class labels for the test data are obtained by mapping soft class scores to real class labels.

## 6. Result

The performance assessment of the models is conducted using evaluation measures such as recall, precision, F1-score, and accuracy. The recall metric quantifies the proportion of correctly predicted positive instances in relation to the combined number of correctly predicted positive and incorrectly predicted negative instances. Precision is a metric that quantifies the proportion of accurate positive predictions relative to the total number of positive forecasts, including both true positive and false positive predictions. The F1-score is a statistic that provides a balanced assessment of model performance by computing the harmonic mean of accuracy and recall. Accuracy is computed as the ratio of true positive and true negative predictions to the sum of true positive, true negative, false positive, and false negative predictions.

The confusion matrix is used to determine the true positive (TP), false negative (FN), true negative (TN), and false positive (FP) values. The number of samples that have been correctly identified as positive, according to the true positive labels, is represented by the variable TP. The number of samples that were predicted to be positive but do not match the actual positive labels is referred to as False Negatives (FN).TN refers to accurately predicted negative samples, and FP indicates samples that were predicted as negative but had actual positive labels. The accuracy metric assesses the proportion of accurately predicted labels in the test data.

### 6.1. Classification of Host by Considering Family Label

The classification problem addressed in this study involves categorizing host labels (bacteria, fungi, humans, plants) from DNA sequences obtained from the NCBI dataset. Among the proposed methods, VCAE-MLELM significantly outperforms SCAE-MLELM in terms of accuracy. This improvement can be attributed to the approach used by VCAE-MLELM, which utilizes the mean and variance of each input feature to predict the output rather than solely relying on the weights of hidden nodes.

**Figure 4:**
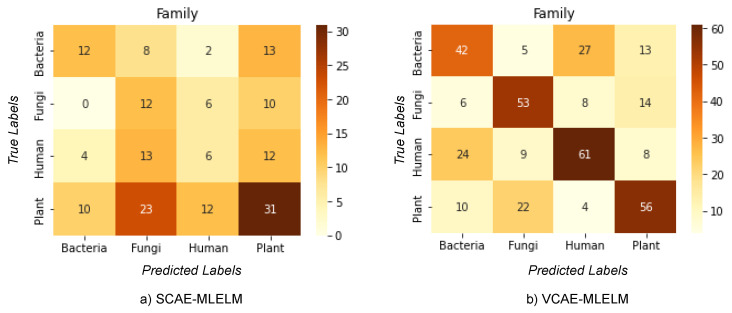
Confusion Matrices of Host label by considering the Family label only. (**a**) SCAE-MLELM generated confusion matrix and (**b**) VCAE-MLELM model generated.

The highest accuracy achieved by the models is 61% for VCAE-MLELM and 31% for SCAE-MLELM, as demonstrated in the confusion matrix shown in Figure 4 and Table 1. It is evident from the confusion matrix that SCAE-MLELM exhibits a higher misclassification rate compared to VCAE-MLELM. This can be attributed to the fact that the number of similar nucleotide groups formed in the sequences is quite low. Furthermore, the presence of unique features at the family class level in the phylogenetic tree further contributes to the high misclassification rate of host labels.

In summary, the VCAE-MLELM model demonstrates superior performance with a higher accuracy rate compared to SCAE-MLELM. Misclassification of host labels remains a challenge, primarily due to the low occurrence of similar nucleotide groups and the unique characteristics of the family class in DNA sequences.

### 6.2. Classification of Host by Considering Family and Class Label

**Figure 5:**
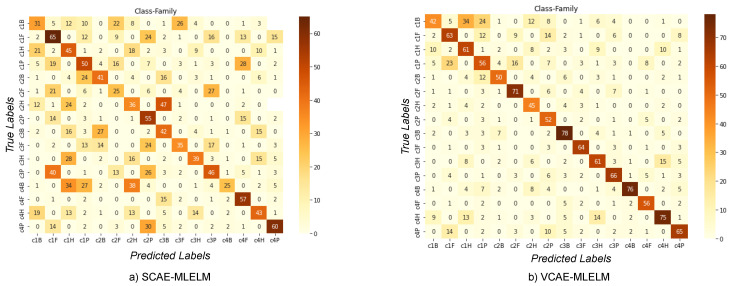
Confusion Matrices of Host label by considering the Family and Class labels. (**a**) SCAE-MLELM generated confusion matrix and (**b**) VCAE-MLELM model generated.

The confusion matrix for both models, VCAE-MLELM and SCAE-MLELM, in the four-category problem is shown in Figure 5. As expected, the VCAE-MLELM model outperforms the SCAE-MLELM model, achieving the highest accuracy rate of 78%. On the contrary, the SCAE-MLELM model only achieves an accuracy rate of 65%, Table 2. Misclassifications are more prevalent in the SCAE-MLELM model, indicating its lower performance.

Although we considered the family label along with the genus and order categories, we chose not to showcase it due to the significantly high rate of misclassification in the SCAE-MLELM model. The genus category, being lower than the family label, does not effectively consider a larger number of nucleotides. On the other hand, the order label considers slightly more nucleotides than the family label, but we focus on the class label.

Analyzing the confusion matrix, we can observe distinct patterns of misclassification more prominently in the SCAE-MLELM model. False predictions are not evenly spread out but rather concentrated towards the left part of the matrix. On the contrary, the VCAE-MLELM model exhibits fewer false classifications.

This analysis further confirms the superior performance of the VCAE-MLELM model compared to the SCAE-MLELM model in the four-category problem. The results highlight the importance of selecting appropriate labels and considering larger nucleotide patterns to achieve accurate classification in DNA sequence analysis.

### 6.3. Classification of Host by Considering Family and Clade Label

**Figure 6:**
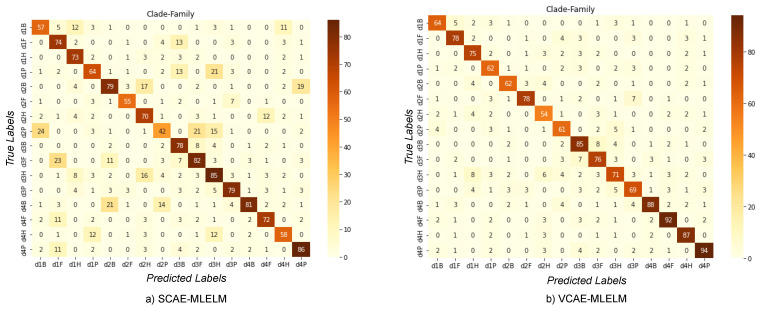
Confusion Matrices of Host label by considering the Family and Class labels. (**a**) SCAE-MLELM generated confusion matrix and (**b**) VCAE-MLELM model generated.

In the four-category problem, we present the confusion matrix (Figure 6) for both the VCAE-MLELM and SCAE-MLELM models, which demonstrates a significantly improved scenario for both models. Consistently, the VCAE-MLELM model outperforms the SCAE-MLELM model, achieving the highest accuracy rate of 94%, Table 3. On the contrary, the SCAE-MLELM model achieves an accuracy rate of 86%. Misclassifications occur at a lower rate for the SCAE-MLELM model.

In our experimentation, we considered the inclusion of the phylum and kingdom categories as well. However, the results for the phylum label were similar to those of the class label, while incorporating the kingdom label resulted in overfitting the model. The inclusion of the kingdom label led to high variance and low error rates.

**Figure 7:**
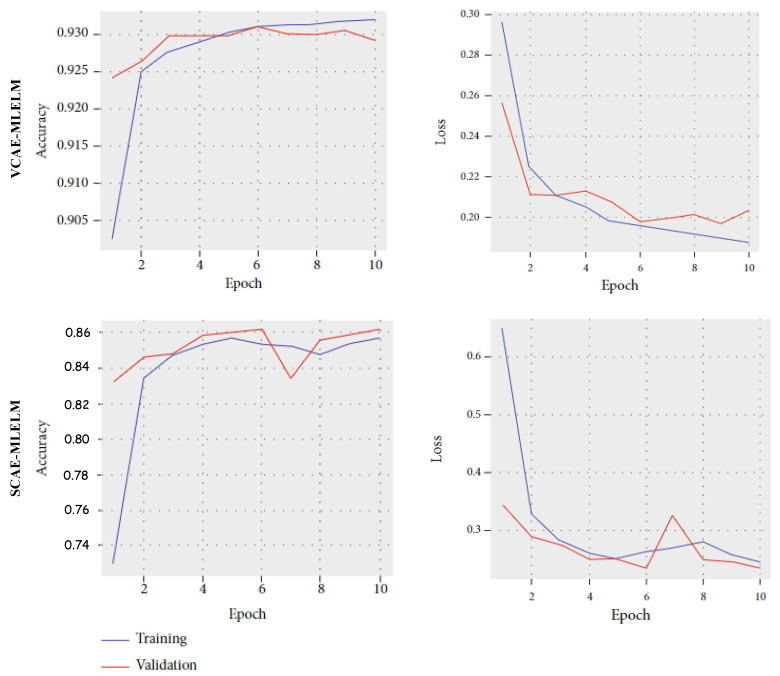
Training and Validation accuracy and loss curve for family-clade label consideration.

To further enhance performance, increasing the number of input data points to the model could be beneficial. Additionally, Figure 7 illustrates the accuracy and loss curves for both proposed models. For the VCAE-MLELM model, the accuracy rate gradually increases until the 10th epoch and then stabilizes. In contrast, the SCAE-MLELM model remains unstable throughout the training phase.

Overall, the results highlight the superior performance of the VCAE-MLELM model in the four-category problem. The findings suggest that further improvements can be achieved by increasing the size of the dataset and carefully selecting relevant labels for classification.

### 6.4. Comparison between Existing Algorithm

To evaluate the effectiveness and efficiency of the system, a comparative study is carried out with two well-known models: CNN-Bidirectional LSTM and DeepMicrobe. This review uses datasets with both picture (2D) and word (1D) inputs with the aim of classifying. As stated in their individual publications, the NCBI dataset was adjusted to meet the model’s requirements. The perspective article provides information on the specific operations of these models as well as the generation of the output. Table 4 shows the aggregate test results in terms of the statistical metrics.

This study examines the individual labeling problems, including family, class, and clade, as well as a combination of multiple labels, such as family-class, family-clade, and class-clade. The highest accuracy is achieved when the family-clade or class-clade labels are taken into consideration by the models. The VCAE-MLELM model demonstrates superior performance compared to other models, achieving a remarkable accuracy rate of 94% when evaluating the family-clade and class-clade labels. The accuracy of the SCAE-MLELM model reaches its peak at 88% when taking into account the class-clade label. The CNN-Bidirectional LSTM and DeepMicrobe models attain their peak accuracy rates of 87% (class-clade) and 84% (family-clade), respectively.

From Table 4, we can observe that the VCAE-MLELM model outperforms the existing models and the SCAE-MLELM model. This can be attributed to the architecture of the VCAE-MLELM model, which incorporates the mean and variance of the extracted features from the convolutional layer in the encoded section before deconvolution.

The proposed models consistently outperform existing algorithms in terms of various metrics, whether using text or image input. This superiority can be attributed to the Multilabel structure of the proposed models, as the existing models exhibit better performance with a single-label data structure.

## 7. Conclusions

This paper aimed to address the challenges of taxonomic classification of DNA sequences by creating a dataset using the publicly available NCBI viral resource database. The data set was constructed by splitting each sequence into segments of 3000 nucleotides with a 50% overlap between DNA sequences of similar species. These sequences were then encoded using one-hot encoding and transformed into numerical arrays using the LabelBinarizer function in Python.

To capture the relationship between K-mer sequences, we utilized a word embedding layer to build a dense feature vector matrix. This matrix preserved the positional information of each K-mer sequence and its neighboring sequences. Two models were proposed for classifying the host of the sequence based on this dense feature matrix. The first model, Stacked Convolutional Autoencoder (SCAE), generated a detailed feature vector that captured label relationships. Subsequently, Multilabel Extreme Learning Machines (MLELM) were used to generate soft classification scores and hard labels from the feature map based on the training data. The second model, the Variational Convolutional Autoencoder (VCAE), calculated the mean and variance of each feature before passing it to the MLELM network for classification. VCAE-MLELM demonstrated superior performance by accurately extracting essential features and achieving higher accuracy compared to SCAE-MLELM.

To evaluate the system’s performance, efficiency, and robustness, extensive training, validation, and testing were conducted. The proposed VCAE-MLELM outperformed existing algorithms such as CNN-Bidirectional LSTM and DeepMicrobe, achieving an accuracy score of 94%, while SCAE-MLELM achieved 88% accuracy. This indicates that the techniques employed in the VCAE-MLELM models are more reliable in recognizing relevant salient features from DNA sequences. The incorporation of MLELM allowed the consideration of two labels at a time during classification, which proved to be effective on the basis of experimentation. Taking into account the family label alone resulted in 31% accuracy for SCAE-MLELM and 61% for VCAE-MLELM. However, considering the combination of family and clade labels, the highest accuracy scores were obtained for both models, reaching 86% for SCAE-MLELM and 94% for VCAE-MLELM. This improvement can be attributed to the analysis of a larger group of 4-mer sequence nucleotides and the examination of more relationships between K-mer sequences, which provided a more accurate probability data space for VCAE. It is worth noting that considering more than two labels led to the overfitting of the models. Therefore, optimal performance was achieved by considering two labels at a time.

## Figures and Tables

**Table 1:** Classification Results for Host by considering “Family” label; precision, recall, and f1-Score by using the SCAE-MLELM and VCAE_MLELM model.

	SCAE-MLELM			VCAE-MLELM		
**Class Group**	**Precision**	**Recall**	**f1-Score**	**Precision**	**Recall**	**f1-Score**
Bacteria	0.08	0.12	0.10	0.37	0.42	0.40
Fungi	0.013	0.12	0.08	0.47	0.53	0.49
Human	0.04	0.06	0.015	0.46	0.61	0.60
Plant	0.025	0.31	0.12	0.55	0.56	0.48
Accuracy	0.31			0.61		

**Table 2:** Classification Results for Host by considering “Family and Class” label; precision, recall, and f1-score by using the SCAE-MLELM and VCAE-MLELM model.

	SCAE-MLELM			VCAE-MLELM		
**Class Group**	**Precision**	**Recall**	**f1-Score**	**Precision**	**Recall**	**f1-Score**
Bacteria	0.34	0.42	0.37	0.64	0.78	0.76
Fungi	0.64	0.65	0.57	0.62	0.71	0.63
Human	0.40	0.45	0.39	0.72	0.75	0.63
Plant	0.57	0.60	0.50	0.63	0.66	0.55
Accuracy	0.65			0.78		

**Table 3:** Classification Results for Host by considering “Family and Clade” label; precision, recall, and f1-score by using the SCAE-MLELM and VCAE_MLELM model.

	SCAE-MLELM			VCAE-MLELM		
**Class Group**	**Precision**	**Recall**	**f1-Score**	**Precision**	**Recall**	**f1-Score**
Bacteria	0.76	0.81	0.73	0.80	0.88	0.86
Fungi	0.74	0.82	0.75	0.84	0.92	0.85
Human	0.80	0.85	0.76	0.83	0.87	0.78
Plant	0.82	0.86	0.79	0.90	0.94	0.91
Accuracy	0.86			0.94		

**Table 4:** Models performance by considering different co-relationships between the Family, Class, Clade, Family-Class, Family-Clade, and Class-Clade labels.

Model Name	Performance	Family	Class	Clade	Family-Class	Family-Clade	Class-Clade
SCAE-MLELM	Accuracy	0.31	0.38	0.46	0.65	0.86	**0.88**
	Precision	0.25	0.29	0.44	0.52	0.60	0.78
	F1-Score	0.35	0.32	0.57	0.42	0.58	0.81
	AUC (%)	0.41	0.42	0.46	0.63	0.75	0.79
	pAUC (%)	0.24	0.37	0.37	0.72	0.68	0.68
VCAE-MLELM	Accuracy	0.61	0.78	0.85	0.78	**0.94**	0.94
	Precision	0.58	0.84	0.77	0.67	0.92	0.89
	F1-Score	0.62	0.82	0.74	0.73	0.87	0.91
	AUC (%)	0.59	0.37	0.49	0.61	0.87	0.90
	pAUC (%)	0.67	0.60	0.68	0.87	0.86	0.78
CNN-Bidirectional LSTM [1]	Accuracy	0.21	0.42	0.48	0.85	0.75	**0.87**
	Precision	0.18	0.35	0.40	0.75	0.44	0.78
	F1-Score	0.19	0.41	0.37	0.68	0.57	0.80
	AUC (%)	0.13	0.28	0.46	0.79	0.60	0.66
	pAUC (%)	0.11	0.16	0.38	0.80	0.68	0.47
DeepMicrobe [2]	Accuracy	0.35	0.48	0.54	0.83	**0.84**	0.84
	Precision	0.40	0.42	0.57	0.82	0.75	0.74
	F1-Score	0.38	0.38	0.62	0.78	0.69	0.65
	AUC (%)	0.24	0.44	0.58	0.75	0.79	0.79
	pAUC (%)	0.32	0.36	0.36	0.68	0.84	0.83

## Data Availability

The datasets utilized in this article were obtained from “NCBI database”, which is freely accessible for all scientists and investigators to conduct experiments and can be accessed through the website: https://www.ncbi.nlm.nih.gov/labs/virus/vssi/#/ (accessed on 25 August 2023).

## Abbreviations

The following abbreviations are used in this manuscript:

A	Adenine
ANN	Artificial Neural Networks
BLAST	Basic Local Alignment Search Tool
C	Cytosine
CNN	Convolutional Neural Network
DL	Deep Learning
DNA	Deoxyribose Nucleic Acid
ELM	Extreme Learning Machine
FN	False Negative
FP	False Positive
G	Guanine
LSTM	Long Short-Term Memory
MLELM	Multilabel Extreme Learning Machine
MSE	Mean Squared Error
NCBI	National Center for Biotechnology Information
NLP	Natural Language Processing
NT	Nucleotide
ReLU	Rectified Linear Unit
RNA	Ribonucleic Acid
SCAE	Stacked Convolutional Autoencoder
SMOTE	Synthetic Minority Oversampling Technique
T	Thymine
TN	True Negative
TP	True positive
U	Uracil
VAE	Variational Autoencoder
VCAE	Variational Convolutional Autoencoder
